# An Economic Evaluation of a Video- and Text-Based Computer-Tailored Intervention for Smoking Cessation: A Cost-Effectiveness and Cost-Utility Analysis of a Randomized Controlled Trial

**DOI:** 10.1371/journal.pone.0110117

**Published:** 2014-10-13

**Authors:** Nicola E. Stanczyk, Eline S. Smit, Daniela N. Schulz, Hein de Vries, Catherine Bolman, Jean W. M. Muris, Silvia M. A. A. Evers

**Affiliations:** 1 Department of Health Promotion, School for Public Health and Primary Care (CAPHRI), Maastricht University, Maastricht, the Netherlands; 2 Department of Communication Science, Amsterdam School of Communication Research/ASCoR, University of Amsterdam, Amsterdam, the Netherlands; 3 Department of Psychology and Educational Sciences, Open University of the Netherlands, Heerlen, the Netherlands; 4 Department of Family Practice, School for Public Health and Primary Care (CAPHRI), Maastricht University, Maastricht, the Netherlands; 5 Department of Health Services Research, School for Public Health and Primary Care (CAPHRI), Maastricht University, Maastricht, the Netherlands; 6 Trimbos Institute, Netherlands Institute of Mental Health and Addiction, Utrecht, the Netherlands; Monash University, Australia

## Abstract

**Background:**

Although evidence exists for the effectiveness of web-based smoking cessation interventions, information about the cost-effectiveness of these interventions is limited.

**Objective:**

The study investigated the cost-effectiveness and cost-utility of two web-based computer-tailored (CT) smoking cessation interventions (video- vs. text-based CT) compared to a control condition that received general text-based advice.

**Methods:**

In a randomized controlled trial, respondents were allocated to the video-based condition (N = 670), the text-based condition (N = 708) or the control condition (N = 721). Societal costs, smoking status, and quality-adjusted life years (QALYs; EQ-5D-3L) were assessed at baseline, six-and twelve-month follow-up. The incremental costs per abstinent respondent and per QALYs gained were calculated. To account for uncertainty, bootstrapping techniques and sensitivity analyses were carried out.

**Results:**

No significant differences were found in the three conditions regarding demographics, baseline values of outcomes and societal costs over the three months prior to baseline. Analyses using prolonged abstinence as outcome measure indicated that from a willingness to pay of €1,500, the video-based intervention was likely to be the most cost-effective treatment, whereas from a willingness to pay of €50,400, the text-based intervention was likely to be the most cost-effective. With regard to cost-utilities, when quality of life was used as outcome measure, the control condition had the highest probability of being the most preferable treatment. Sensitivity analyses yielded comparable results.

**Conclusion:**

The video-based CT smoking cessation intervention was the most cost-effective treatment for smoking abstinence after twelve months, varying the willingness to pay per abstinent respondent from €0 up to €80,000. With regard to cost-utility, the control condition seemed to be the most preferable treatment. Probably, more time will be required to assess changes in quality of life. Future studies with longer follow-up periods are needed to investigate whether cost-utility results regarding quality of life may change in the long run.

**Trial Registration:**

Nederlands Trial Register NTR3102

## Introduction

Web-based smoking cessation interventions have large potential for public health [1], [2]. The enormous increase in the use of the Internet has made such interventions increasingly common all over the world [3]. Different web-based computer-tailored (CT) smoking cessation interventions have already been developed and implemented to aid people to quit smoking [1], [4], [5]. Previous research has indicated that tailored health messages were successful in attracting smokers' attention, resulting in an enhanced processing of the health message [6], [7], and an increase in smoking cessation rates [8], [9].

Even though evidence exists for the effectiveness of web-based CT smoking cessation interventions [1], [5], [8], they often have difficulty attracting and holding respondents [10]–[12]. Most of the web-based CT interventions consist of simple text messages [1], [5], [8]. However, since websites increasingly make use of new web-based technologies like videos or pictures, simple text messages may no longer be attractive enough for current Internet users. Less educated groups, in particular, often quit web-based interventions before completing all elements of the intervention [13], [14]. Since video-based messages seem to require less mental effort and may help the person to concentrate on the core elements of the message [15], the integration of video messages may increase the appeal and potentially the (cost-)effectiveness of web-based CT smoking cessation interventions. As a result, two multiple CT smoking cessation interventions were developed: 1) a text-based multiple CT intervention, where smokers received tailored messages via text and 2) a video-based multiple CT smoking cessation intervention, where smokers received tailored messages via video [16].

Although web-based smoking cessation interventions have already demonstrated favourable behaviour change outcomes [1], [2], to date relatively little is known about whether these CT interventions are preferable in terms of their cost-effectiveness, when compared to other treatments. Given the fact that health-care budget holders have to make choices regarding the implementation of different smoking cessation programmes, it is essential to know whether the societal benefits of these programmes are worth the investments that have to be made to offer them [17]. To date, several economic evaluations of smoking cessation interventions have already been conducted [18]–[22]. The majority of these studies reported that these interventions were cost-effective since they were relatively low in costs accounting for the resulting gains in terms of avoided mortality and the prevention of care costs for smoking-related diseases. To our current knowledge, only one recent study in the Netherlands has investigated the cost-effectiveness and cost-utility of a web-based CT smoking cessation intervention [23]. This study showed that a web-based CT smoking cessation intervention seemed to be more cost-effective with regard to smoking abstinence rates assessed after twelve months than the usual smoking cessation care in the general practice setting, taking into account a willingness to pay of €18,000. However, the cost-utility analysis (calculating utility outcomes in monetary values) revealed that care as usual was the most efficient treatment [23]. Even if web-based smoking cessation interventions are found to be as effective as, or less effective than traditional interventions (e.g. face-to-face counselling or care as usual), their possible lower delivery costs via the Internet may result in interventions being more cost-effective [3]. As web-based interventions have the potential to contribute to reducing health-care and societal costs, economic evaluations of these interventions are required to be able to choose the most cost-effective treatment.

Given the importance of this topic, the purpose of the current study was to compare the cost-effectiveness and cost-utility of 1) a video-based multiple CT smoking cessation intervention, 2) a text-based multiple CT smoking cessation intervention, and 3) a control condition (respondents received brief general text advice about quitting) after a follow-up period of twelve months.

## Methods

### Design and participants

**Figure 1:**
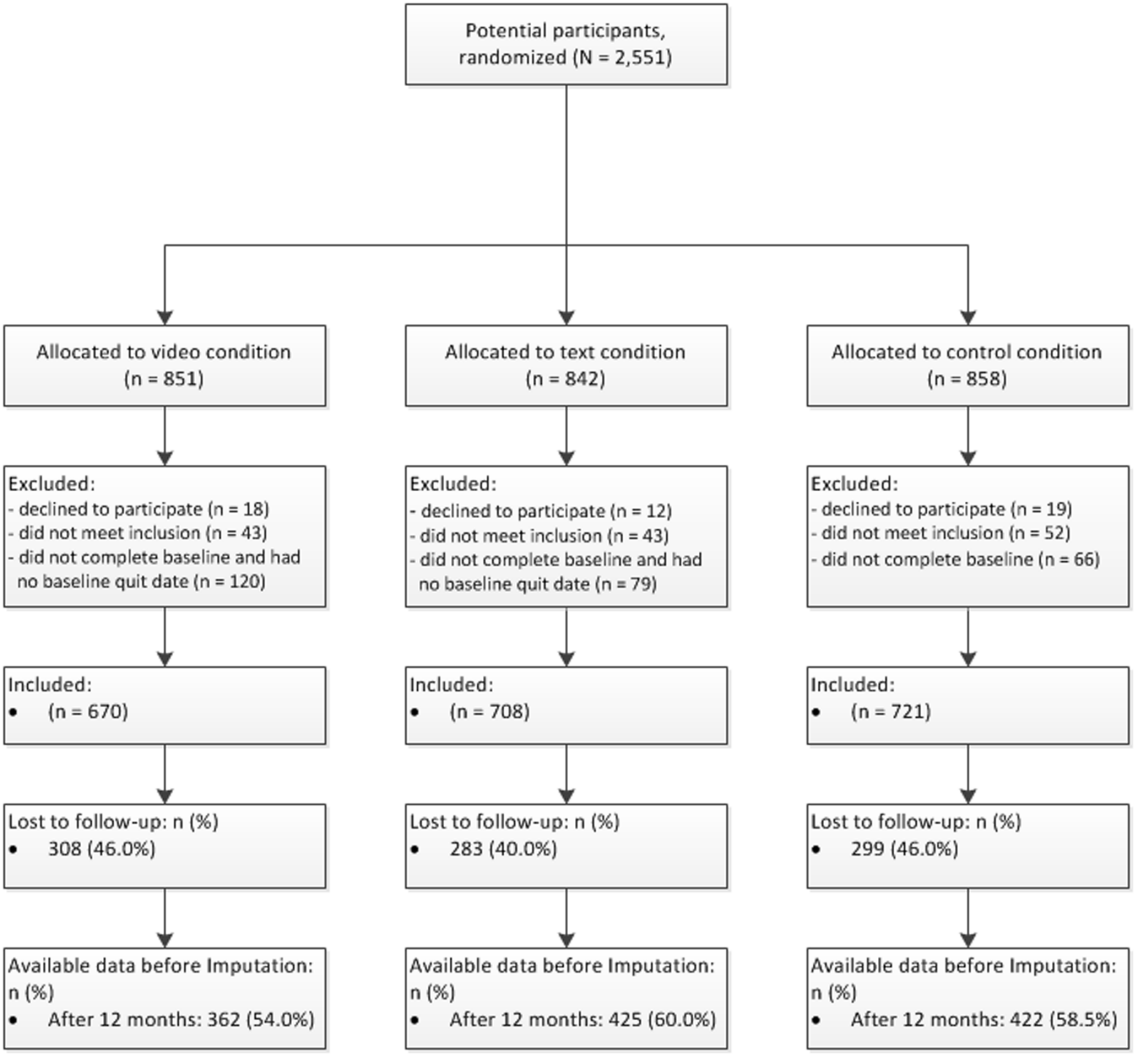
Flowchart of respondents.

The current economic evaluation study was embedded in a three-group randomized controlled trial (RCT) that tested the effectiveness of two multiple CT smoking cessation interventions against a control condition. The study was submitted for approval to the Medical Research Ethics Committee (MREC) of the Atrium Medical Centre Heerlen, the Netherlands. The MREC decided that no MREC approval was needed for this study because respondents were not obliged to engage in a certain act. Additionally, the questionnaires of the intervention were judged not to have a deep psychological impact. Nonetheless, when submitting our study protocol for publication we were advised to register the trial and we did so shortly after the enrolment of respondents had started. We can thus confirm that all ongoing and related trials for this intervention are registered at the Dutch Trial Register (NTR3102) (http://www.trialregister.nl/trialreg/admin/rctview.asp?TC=3102) [24]. The study was in line with the ethical codes of conduct of the American Psychological Association (APA) [25]. Dutch smokers were included to participate in the RCT from December 2010 up to June 2012 by general practices throughout the Netherlands, several Dutch paid advertising campaigns and different health funds. Interested smokers could receive more information on the website and could sign up via the study website (http://www.steunbijstoppen.nl) [26] by choosing their own username and password. This website was accessible on the Internet, so smokers could choose to sign up at home, at work, or anywhere else where they had access to the Internet. Smokers were randomly allocated to one of the two experimental conditions (video- vs. text-based CT) or the control condition. Randomization took place via a computer software device [27]. After giving online informed consent, respondents were included when they were daily smokers, 18 years or older, motivated to quit smoking within the following six months and had Internet access. The flow chart of respondents is presented in Figure 1. The trial had follow-up measurements after six (between May 2011 and January 2013) and twelve months (between December 2011 and June 2013). The protocol for this trial and supporting CONSORT checklist are available as supporting information; see Checklist S1 and Protocol S1.

### Intervention

**Figure 2:**
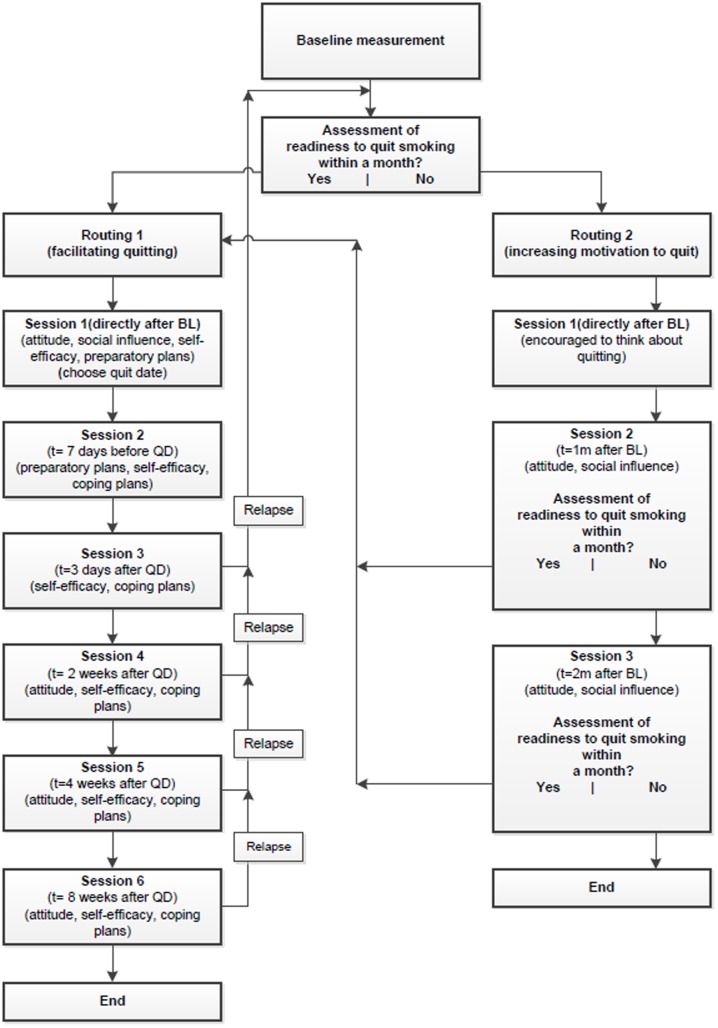
Intervention design. Legend: T = time, BL = Baseline, QD =  Quit date, 1 m = 1 month, 2 m = 2 months.

The two web-based multiple CT smoking cessation interventions were based on two earlier tested CT interventions that have been shown to be effective [8], [9]. The I-Change model formed the theoretical framework of the two interventions. After filling out the baseline measurement, respondents received tailored feedback on their smoking behaviour, attitude, perceived social influence, perceived self-efficacy, and on how to plan a quit date. At the end of the session, respondents were asked to set a quit date within the following month. Depending on respondents' readiness to quit smoking within the following month, they received personalized feedback during multiple CT sessions and were directed into one of two routings (see Figure 2).

#### Routing 1

The aim of routing 1 was to help smokers to facilitate the translation of their intention into action by providing tailored feedback to increase self-efficacy and effective action planning. Respondents ready to quit within one month were directed to routing 1, which consisted of six different sessions: 1) at baseline, after feedback on their smoking behaviour, their attitude, social influence and self-efficacy, respondents were asked to choose a quit date; 2) one week before their quit attempt, respondents received tailored feedback on the extent to which they had planned their quit attempt (preparatory plans), on their perceived self-efficacy to deal with difficult situations and on how to cope with these situations (coping plans); 3) three days after their quit attempt, respondents received feedback on their perceived self-efficacy, including feedback on how to deal with difficult situations and were invited to formulate coping plans to prevent potential relapse; 4, 5 and 6) two, four and eight weeks after their quit date, respondents received tailored feedback on their perceived self-efficacy, on how to deal with difficult situations and on their attitude towards smoking and quitting smoking. During these last three sessions, respondents could choose to receive feedback on different items. This option was provided since it was expected that they would encounter different problems during their quit attempt.

#### Routing 2

The goal of routing 2 was to quit smoking and to increase motivation by increasing perceptions of the pros of quitting and how to obtain support for quitting. Respondents who were not ready to quit within one month were directed to routing 2, which including several sessions: 1) directly after baseline, respondents were encouraged to use the next month to reflect on their motivation to quit smoking; 2) one month after baseline, respondents received tailored feedback on their smoking behaviour, their attitude regarding smoking and quitting smoking, their perceived social support and their readiness to quit smoking; respondents ready to quit within a month were directed to routing 1; 3) two months after baseline, respondents who were still not ready to quit received another tailored feedback letter, using a similar strategy as used before in session 2; respondents who were ready to quit within one month were directed to routing 1. If respondents were still not prepared to quit, they received an email telling them that the programme would end at this stage and that they would receive no further invitations.

After each feedback moment (in routings 1 and 2), a summary with all tailored feedback messages was sent to the respondent by email. A detailed description of the study design can be found elsewhere [16].

### Mode of delivery strategy

In the text-based CT intervention, respondents received text-based CT messages (without any graphics or animations). In the video-based multiple CT intervention, the same tailored messages were presented as in the text-based intervention; however, the tailored messages were translated from text into videos. The tailored messages were presented by five different adults in a TV ‘news programme’ format.

## Measurements

Self-reported online questionnaires were used to assess effects and costs. Measurements were conducted at baseline, six- and twelve-month follow-up. Respondents who did not complete the follow-up measurements after one and two weeks were prompted twice more by email to complete the questionnaire on the intervention website. Respondents who did not react to the two reminders received another invitation by email to briefly indicate their current smoking status. In this third email, they were asked to fill out a shortened version of the six-and twelve-month follow-up measurement, consisting of 10 important smoking-related questions, which they could send back by email. Lastly, when the follow-up measurement was still not completed, respondents were contacted by a short phone call, with the same questions being asked as in the shortened questionnaire.

### Baseline measurement

The baseline measurement assessed factors related to smoking cessation and societal costs. These included demographics (age, gender, educational level), health-care-related costs and productivity costs (see below), occurrence of smoking-related diseases, and smoking-related factors such as addiction level, which was measured by the Fagerström test for cigarette dependence (FTCD) [28], [29].

### Cost measures

The present economic evaluation was conducted from a societal perspective.

Intervention costs, health-care-related costs, productivity costs and respondent costs were assessed at baseline and at the follow-up measurements, using three-month retrospective questionnaires.

*Intervention costs* included costs that could be attributed to the delivery of the intervention, such as hosting costs for the web-based CT smoking cessation intervention. Costs for the development of the intervention and research-specific costs were excluded since these costs are non-recurring costs that will not be spent again if the intervention is implemented elsewhere.

*Health-care-related costs* related to general practitioners' or practice nurses' consultations or home visits, inpatient and outpatient specialist care, mental health care, alternative medicine, hospital admissions, smoking cessation aids, prescribed and over-the-counter medication, and other care (e.g. professional home care, paramedic consultations). Respondents had to indicate what type of care they had received and how often they had received it during the previous three months. The updated Dutch manual for cost analysis in health-care research was used to value the health-care-related costs of the respondents [30]. If costs for medical health care were not found in the manual, costs were searched on the Internet. Normally, the mean price of three different cost prices was used. In the case of uncertainty, the lowest cost price was taken. Furthermore, the Dutch website www.medicijnkosten.nl was used to value medication costs. The costs of medications were calculated based on the dose described by the respondent, i.e. costs per tablet, gramme or millilitre were used to calculate the total medication costs for each respondent.

*Productivity costs* related to absenteeism. Productivity losses for paid work were calculated according to the human capital method using a mean income valuation of the Dutch population [30]. This approach estimates the annual earnings for each year of potential lost employment and adds these earnings together. Since income differs between age and gender, productivity losses were valued differently based on respondents' age and gender [30].

*Respondent costs* consisted of costs related to the time respondents spent on the CT intervention and travel costs. For the time spent on the intervention, we used a mean score that was needed to complete all intervention elements (without the follow-up measurements). A mean score of 120 minutes was used for the two CT interventions (video vs. text CT) and five minutes for the control condition. To determine the costs regarding time spent on the website, we used the human capital method using the mean valuation of the Dutch population (again, costs were valued differently based on age and gender) [30]. Travel costs were calculated based on the mean costs per kilometre and the average distance to healthcare providers in the Netherlands [30].

### Indexing costs

Cost prices of the current study were from the years 2009–2013 and were therefore indexed to the year 2013. The consumer price indices used were 105.38 for the year 2009, 106.72 for the year 2010, 109.22 for the year 2011, 111.90 for the year 2012 and 115.00 for the year 2013 [31]. Since the follow-up period was twelve months, discounting for differential timing was not necessary [23].

### Outcome measure cost-effectiveness and cost-utility

Cost-effectiveness analysis shows the effects of alternative interventions in units that are relevant to the condition (e.g. in our case smoking abstinence) and the costs of these interventions in monetary units. It provides information about the relative efficiency of alternative interventions that aim at the same goal (e.g. smoking cessation). The benefit of our cost-effectiveness analysis is that it allows policy makers to compare the net benefit of the intervention to other smoking cessation interventions.

Cost-utility analysis on the other hand is a form of cost-effectiveness analysis that aims to calculate the cost per unit of utility. A common unit of utility is quality adjusted life-years (QALY). The benefit of cost-utility analysis is that its results allow comparing outcomes of economic evaluations across diverse other health-related interventions, also (having other goals than smoking cessation, e.g. improvements in dietary behaviour or physical activity) [32], [33].

The primary outcome measure for the cost-effectiveness analysis (CEA) was prolonged abstinence (PA), which was assessed by one item asking respondents whether they had refrained from smoking (including a grace period of two weeks where someone could have smoked a maximum of 1–5 cigarettes) since their last quit attempt (1 = yes; 0 = no). In line with the definition of PA, those who reported that they had quit less than nine months before the follow-up measurements were not included as quitters in the PA measurement [34]. A secondary outcome measure for the CEA was seven-day point prevalence abstinence (PPA), which was assessed by one item asking respondents whether they had refrained from smoking during the last seven days (1 = yes; 0 = no).

The outcome measure for the cost-utility analysis (CUA) was quality of life, measured in terms of quality-adjusted life years (QALYs). In order to estimate the effects in QALYs, utility scores needed to be calculated. Utilities can be regarded as the preferences that society has for a set of health-related outcomes [35]. In the current study, health states were assessed by the Dutch version of the Euro-Qol (EQ-5D-3L) which is regarded as a validated measure to assess quality of life and which is often used in the Netherlands [35]–[37]. The Dutch EQ-5D is a measure of self-reported health outcomes that is applicable to a wide range of health conditions. The EQ-5D-3L entails five health state dimensions: mobility, self-care, usual activity, pain/discomfort, and anxiety/depression. Each dimension was assessed by asking respondents to specify their health status, resulting in three-point scale (3 = many complaints, 2 = some complaints, 1 = no complaints) [38]. The health states, coming from the Euro-Qol questionnaire, were transformed into a utility score, using the Dutch tariff [39]. Utility scores could range from −0.33 (worst imaginable health status) to 1 (perfect health status). The utility scores of the three measurements (baseline, and six- and twelve-month follow-up) were computed into an overall QALY score, whereby the ‘area under the curve method’ was used. The area under the curve is regarded as the duration of the health state, i.e. survival (on the x-axis; one year) multiplied by the quality weight for the health state (y-axis; QALYs gained). In this case, the number of QALYs represents the life years gained during a one-year follow-up measurement [35].

## Analyses

### Baseline characteristics and dropout

To investigate possible differences between the three conditions with regard to demographics, baseline values of the outcomes and societal costs over the previous three months, Chi-square tests and one-way analyses of variance (ANOVAs) were conducted. If Chi-square or ANOVA tests showed a p<.05, post-hoc pairwise comparisons were performed. Logistic regression analysis was used to explore whether there were differences between those lost to follow-up and those who remained in the study after twelve months follow-up.

### Imputation

During this study, missing data for costs and effects were imputed using several strategies that were also used in previous cost-effectiveness studies [18], [23], [40]. Missing data for the costs, EQ-5D-3L questions and smoking related variables were first replaced by mean imputation, using respondents' scores on the previous and next measurements. If we were not able to impute missing data by mean imputation, we replaced our missing data by using the last observation carried forward technique (LOCF). Mean imputation followed by LOCF is a common approach to replace missing data in trial-based cost-effectiveness analyses with incomplete observations [19], [23], [40]. Although LOCF is considered to be conservative it has been argued to be a better option than using observed data only, a strategy also referred to as complete case analyses [41]. Unrealistic values (e.g. more than 150 meetings with a psychologist during the last three months) were replaced with the highest realistic value, a strategy also used previously [42]. The same methods were used to impute missing cost data for the six-and twelve-month measurement. For missing effect data, i.e. missing data for the smoking abstinence variables, a negative scenario was used to replace missing values, in which respondents who did not fill in the follow-up measurement were regarded as smokers. This ‘negative’ scenario is recommended by the Russell standard and was chosen to avoid a false positive intervention effect [43].

### Costs and effects at the twelve month follow-up

To deal with uncertainty around the estimates of cost-effectiveness and cost-utility, mean costs of the three groups were compared using nonparametric bootstrapping (5000 times) with 95% confidence intervals in percentiles [35]. One-way ANOVAs and Chi-square tests were conducted in order to investigate whether any differences existed between the groups regarding costs and effects after twelve months follow-up.

### Cost-effectiveness & cost-utility analyses

In order to compare the cost-effectiveness and cost-utility of the three conditions, an incremental cost-effectiveness ratio (ICER) was calculated (probability of smoking abstinence/QALYs). For quality of life, ICERs are often called incremental cost-utility ratios (ICURs). However, since ICERs and ICURs can only compare two groups, a net monetary benefit (NMB) had to be calculated in order to compare the cost-effectiveness and cost-utility of the three conditions in our study [44]. The NMB was calculated by valuing the effectiveness and utility outcomes in monetary values using a threshold for society's willingness to pay (WTP). In the Netherlands, cut-off points for preventive interventions have been established for the WTP, varying from €0 to €80,000 [45]. A WTP of €18,000 is an accepted Dutch cut-off point for the WTP per QALYs [45]; therefore, for both outcome measures (smoking abstinence and QALY) we present the monetary threshold, ranging from €0 to €18,000 to €80,000.

### Sensitivity analyses

In order to deal with uncertainty around the estimates of cost-effectiveness and cost-utility, a nonparametric bootstrap resampling technique was used with 1,000 replacements. Random samples were drawn from the original dataset resulting in 1,000 different samples and accompanying ICERs [35], [46]. Percentages were calculated so that the 1,000 slightly different ICERs resulted in a certain outcome. Four different outcomes were possible: 1) more effects and lower costs (dominant); 2) fewer effects and lower costs; 3) more effects and higher costs; or 4) fewer effects and higher costs (inferior). The resulting decision uncertainty is graphically presented by a cost-effectiveness acceptability curve (CEAC) and a cost-utility acceptability curve (CUAC). In addition, different sensitivity analyses were executed to control for possible uncertainty of parameter estimates during the primary analyses: (1) we used seven-day PPA as outcome variable (instead of PA); (2) we excluded all surgery costs since these costs were extremely high compared to the other health-care-related costs; (3) we tested the results from a health-care perspective and excluded the respondent costs and productivity costs because these might be reflected in the respondents' reported quality of life [30]; (4) we excluded all medication costs, because these were very high compared to the other societal costs.

Data were analyzed using SPSS 19.0 (SPSS, Inc., Chicago, IL. USA). Microsoft Office Excel 2010 was used for all bootstrap analyses.

## Results

### Baseline characteristics

Of the 2,099 respondents who were eligible for the current study, 670 were randomized to the video-based condition, 708 to the text-based condition and 721 to the control condition. Table 1 shows the baseline characteristics of respondents in each of the three conditions, as well as their health-care-related costs and productivity costs over three months prior to baseline. Respondents had a mean age of 45.7 years (SD 12.8), 1,278 (60.9%) of them were female and 705 (33.6%) had a lower level of education. No significant baseline differences were found between the three groups.

**Table 1:** Comparability of the video-, text-based and control condition regarding demographics, baseline values of outcomes and health-care-related costs over the last three months (N = 2099).

Variable	Video (N = 670)	Text (N = 708)	Control (N = 721)	*F*(df)	X^2^(df)	P
Gender [% female (N)]	62.2(417)	60.9(431)	59.6(430)		.985 (2)	.611
Age [M (SD)]	45.54(13.0)	45.42(12.8)	46.2(12.5)	0.770(2, 2096)		.463
Educational level [% (N)]					3.978(4)	.409
High	33.6(225)	32.6(231)	34.5(249)			
Medium	36.9(247)	36.0(255)	38.8(280)			
Low	29.6(198)	31.4(222)	26.6(192)			
FTCD score a (1–10) [M (SD)]	5.0(2.3)	4.9(2.4)	4.9(2.5)	0.774(2, 2096)		.461
Number of cigarettes smoked per day [M (SD)]	19.0(8.1)	18.7(8.4)	19.0(9.2)	0.286(2, 2096)		.751
With COPD diseases [% (N)]	14.5(97)	14.0(99)	13.0(94)		0.630(2)	.730
With cancer [% (N)]	1.5(10)	1.3(9)	2.1(15)		1.568(2)	.457
With diabetes [% (N)]	4.0(27)	4.7(33)	5.4(39)		1.477(2)	.478
With cardiovascular diseases [% (N)]	9.4(63)	8.5(60)	12.1(87)		5.515(2)	.063
With asthma diseases [% (N)]	9.4(63)	8.1(57)	7.1(51)		2.532(2)	.282
Utility score b [M (SD)]	0.8(0.2)	0.8(0.2)	0.8(0.2)	0.271(2, 2096)		.762
Total health-care-related costs (€) c [M (SD)]	756.3(1742.9)	836.4(2509.9)	793.0(2067.1)	0.239(2, 2061)		.788
Prescribed and OTC (€) d medication [M (SD)]	69.3(263.5)	66.7(262.9)	78.0(325.7)	0.301(2, 2062)		.740
Hospital (€) [M (SD)]	166.2(812.8)	252.2(1779.8)	185.0(1044.6)	0.863(2, 2096)		.422
Surgery (€) [M (SD)]	284.7(1022.1)	257.7(985.7)	249.2(968.2)	0.241(2, 2096)		.786
Health-care provider (€) [M (SD)]	230.7(476.3)	245.4(567.4)	274.6(867.1)	0.796(2, 2095)		.451
Productivity costs (€) [M (SD)]	718.9(2664.2)	772.5(2856.5)	835.0(3165.5)	0.279(2, 2095)		.757
Travel costs (€) [M (SD)]	7.8(15.2)	7.6(13.8)	8.3(17.6)	0.249(2, 2095)		.780

a Fagerstroem Test for Cigarette Dependence (0 =  not addicted, 10 =  highly addicted),

b based on the Dutch algorithm for the EQ-5D-3L scores,

c costs for prior three month,

d OTC: over-the-counter.

During this study, 362 out of 670 (54.0%) were followed-up after 12 months in the video condition, versus 425 (60%) out of 708 in the text condition and 422 (58.5%) out of 721 in the control condition. Dropout analyses showed that study condition had no influence on respondents' likelihood of dropping out of the study. These analyses did show, however, that respondents were more likely to remain in the study when they were older (OR = 1.016, *p* = .000) and, when they had the Dutch nationality (OR = 1.624, *p* = .023).

After the imputation of missing values, total cost data were available for 2,082 respondents (99.2%), whereas effect data were available for 2,099 respondents (100%) for the two abstinence measures (PA and PPA) and 2,088 (99.5%) for the QALYs.

### Costs and effects at the twelve months follow-up

Table 2 shows the mean societal costs for the three conditions over a period of one year. Since the experimental conditions were the most time-intensive conditions, the time costs were significantly higher than in the control condition. No significant differences were found between the three conditions with regard to health-care-related costs and productivity costs. Furthermore, no significant group differences were found in QALYs (Table 3).

**Table 2:** Mean annual costs a per respondent in the video-, text-based and control condition.

Cost type	Mean costs (€) (SD) a			95% CI Differences b		
	Video	Text	Control	Video-Text	Control-Text	Video- Control
**Intervention costs** (N = 2099)	0.22	0.22	0.22			
**Total health-care-related Costs (N = 2082)**	4845(509)	5279(532)	4837(464)	−1860.5 to 1018.8	−1824.6 to 948.6	−1317.1 to 1424.7
Prescribed and OTC medication (N = 2082)	310(50)	422(102)	286(45)	−359.8 to 91.5	−372.6 to 60.3	−107.0 to 159.5
Hospital (N = 2099)	1026(319)	895(240)	697(135)	−582.7 to 1003.2	−760.0 to 313.7	−225.9 to1126.0
Surgery (N = 2099)	127(46)	172(58)	143(45)	−197.1 to 95.5	−180.4 to108.9	−142.4 to112.9
Health care provider (N = 2086)	909(95)	1033(98)	1041(119)	−389.4 to 143.8	−293.4 to 313.9	−431.6 to 158.0
**Productivity costs** (N = 2099)	2247(334)	2372(350)	2452(389)	−1064.9 to 824.9	−921.64 to 1114.7	−1202.1 to 804.8
**Total respondents costs (N = 2094)**	97(3)	100(2)	35(2)	−9.8 to 3.7	−71.8 to 59.1	55.3 to 69.6
Travel costs (N = 2099)	31(3)	33(2)	33(2)	−8.6 to 5.2	−6.4 to 6.4	−8.9 to 5.5
Time costs (fixed)	67(1)	67(1)	2(0)	−2.1 to 0.87	**−66.4 to −64.4**	**63.8 to 65.8**

a Volumes and prices details are available upon request,

b based on 5000 bootstrap replications.

**Table 3:** Mean annual effect on smoking abstinence and QALY in the video-, text-based and control condition (intention to treat).

Effects	Video (N = 670)	Text (N = 708)	Control (N = 721)	*F*(df)	X^2^(df)	P
Prolonged abstinent (N = 2099)[% (N)]	9.9(66)	7.3(52)	6.4(46)		6.134(2)	.047
7-day point prevalence abstinent (N = 2099) [% (N)]	17.8(119)	17.7(125)	16.2(117)		.730(2)	.694
QALY (EQ-5D-3L) a (N = 2088) [M (SD)]	0.8(0.2)	0.8(0.2)	0.8(0.2)	.017(2, 2085)		.984

a Based on the Dutch algorithm for EQ-5D-3L scores.

A significant difference was found between the three conditions regarding the effects of the interventions on prolonged abstinence (X^2^ = 6.134, df = 2, *p* = .047) (Table 3). Pairwise comparisons between the different groups revealed that the video-based condition significantly differed from the control condition (*F* (2, 2096) = 3.071; *p* = .047). A higher proportion of respondents in the video-based condition achieved PA than in the control condition.

### Cost-effectiveness and cost-utility analyses

Table 4 presents the incremental costs and effects per prolonged abstinent smoker and per QALY gained. Analyses showed that the video-based condition resulted in slightly higher costs, but also in more effects. From a WTP of €1,500 or higher, the video-based condition appeared to be more likely to be cost-effective than the control condition. From a WTP of €50,400 or higher, the text-based condition seemed to be more cost-effective than the control condition. When costs and effects were compared between the two experimental conditions (video- vs. text-based CT), the video-based condition dominated the text-based condition. Costs in the text-based condition were higher, whereas effects were lower than in the video-based condition.

**Table 4:** Incremental costs and effects per abstinent smoker and per QALY gained for the video-, text-based and control condition with a willingness-to-pay threshold of €18,000.

Intervention					
**Prolonged abstinence** a	**Costs per respondent**	**Abstinence**	**Incremental costs (€)**	**Incremental abstinence** b	**Incremental costs** c
Control	4,879	1.06	-	-	-
Video vs. Control	4,939	1.10	60	0.04	1,500
Text vs. Control	5,383	1.07	504	0.01	50,400
Video vs. Text			444	−0.03	dominated d
**QALY (EQ-5D-3L)** e	**Costs per respondent**	**Quality of life**	**Incremental costs (€)**	**Incremental quality of life**	**Incremental costs per QALY**
Control	4,879	0.83	-	-	-
Video vs. Control	4,939	0.83	60	0.001	60,000
Text vs. Control	5,383	0.83	504	−0.002	dominated
Video vs. Text			444	−0.003	dominated

a coded as 2 =  prolonged abstinent and 1 not prolonged abstinent,

b incremental number of abstinence/QALY,

c per abstinent respondent, calculated according to the formula ICER/ICUR = (Ci-Cc)/Ei-Ec),

d dominated =  less costs, more effects compared to the other condition,

e based on the Dutch algorithm for the EQ-5D-3L scores.

**Figure 3:**
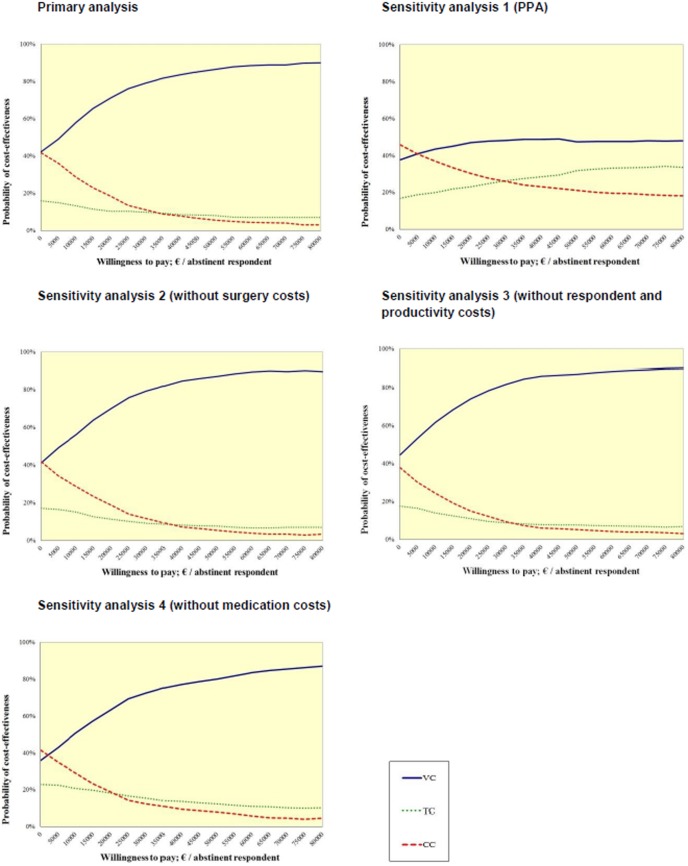
Primary and sensitivity analyses of cost-effectiveness acceptability curve for the three conditions: video-based, text-based and control condition.

The probability that the video-based condition was cost-effective at a WTP of €0.00 per abstinent respondent was 42%, for the text-based condition 16% and for the control condition 42%. With a WTP of €18,000 per abstinent respondent, the video-based condition would probably be the most cost-effective (i.e. 70%), followed by the control condition (i.e. 20%) and the text-based condition (i.e. 11%). With increasing the threshold value up to €80,000, again the video-based condition was the most effective treatment, followed by the text-based condition (i.e. 7%) and control condition (i.e. 3%). The probability of each treatment being more cost-effective than the two other treatments is also presented in the CEAC (Figure 3). In secondary analyses, when seven-day PPA was used as outcome measure (Table 5 & Figure 3), comparable results were found. Here again, the video condition was the most cost-effective treatment (i.e.>38%,>46% and>48% with a WTP of €0.00, €18,000 and €80,000, respectively). All other sensitivity analyses yielded a similar pattern (Table 5 & Figure 3).

**Table 5:** Results from cost-effectiveness and cost-utility analyses based on 1000 bootstrap replications.

Analysis type	Group (N)			Probability of highest net monetary benefit (WTP = 0),%			Probability of highest net monetary benefit (WTP = 18,000),%			Probability of highest net monetary benefit (WTP = 80,000),%		
	Video	Text	Control	Video	Text	Control	Video	Text	Control	Video	Text	Control
**Primary analysis**												
Prolonged abstinence (PA) a	670	708	721	42	16	42	70	11	20	90	7	3
QALY (EQ-5D-3L) b	667	704	717	38	18	45	39	18	43	41	20	39
**Sensitivity analysis**												
Seven-day point prevalence abstinence (PPA) c	670	708	721	38	17	46	46	22	31	48	34	18
PA without surgery costs	670	708	721	41	17	42	68	12	20	90	7	3
PA without respondent costs	670	708	721	43	20	38	68	14	17	90	7	3
PA without medication costs	670	708	721	36	23	42	61	19	21	87	10	4
QALY without surgery costs	667	704	717	43	15	41	44	16	41	44	17	39
QALY without respondents costs	667	704	717	41	18	41	43	18	39	45	18	37
QALY without medication costs	667	704	717	35	27	38	38	26	37	40	24	36

a Coded as 2 =  prolonged abstinent and 1 =  not prolonged abstinent,

b based on the Dutch algorithm for the EQ-5D-3L scores,

c coded as 2 =  seven-day point prevalence abstinent and 1 =  not seven-day point prevalence abstinent;

Results from the cost-utility analyses showed that the text-based condition was dominated by the control condition, since this treatment was less effective and more expensive (Table 4). Furthermore, results revealed that the video-based condition was more expensive and more effective than the control condition in increasing the number of QALYs gained. When comparing the video-based condition with the text-based condition, costs were lower and effects were higher regarding QALYs gained within the video condition. Therefore, the text-based condition was dominated by the video-based condition.

**Figure 4:**
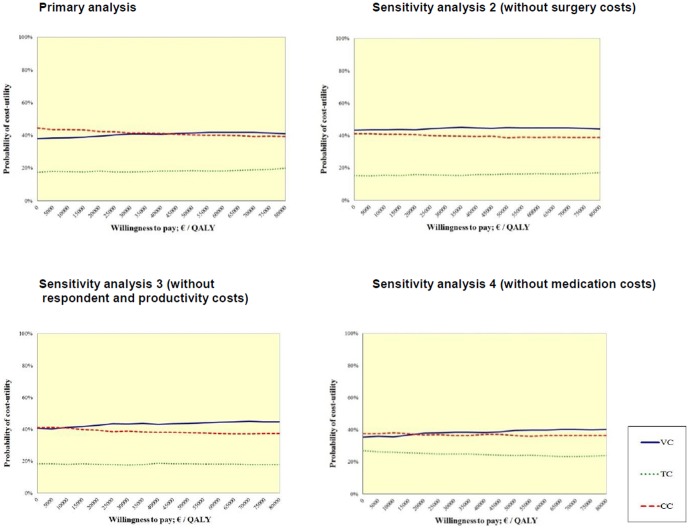
Primary and sensitivity analyses of cost-utility acceptability curve for the three conditions: video-based, text-based and control condition.

The probability that the control condition was efficient at a WTP of €0.00 per QALY was 45%, for the video-based condition 38%, and for the control condition18%. With a WTP of €18,000 per QALY, the control condition would probably be the most efficient treatment (i.e. 43%), followed by the video-based condition (i.e., 39%) and the text-based condition (i.e. 18%). However, increasing the threshold value up to €80,000, the video-based condition would probably be the most efficient treatment (i.e. 41%), followed by the control condition (i.e. 39%) and text-based condition (i.e. 20%). All sensitivity analyses showed a similar pattern (see Table 5). The CUAC for primary and sensitivity analyses are presented in Figure 4.

## Discussion

### Main findings

Until now, there have been only a limited number of economic evaluations of web-based smoking cessation interventions conducted from a broad societal perspective [23]. In addition, even less is known regarding the relative cost-effectiveness and cost-utility of different delivery methods for web-based CT smoking cessation interventions [47]. Therefore, the aim of this study was to investigate the cost-effectiveness and cost-utility of two CT smoking cessation interventions (video- vs. text-based CT), delivered via the Internet. The results of the present study revealed that the video-based CT smoking cessation intervention was the most cost-effective treatment for smoking abstinence assessed after twelve months from a willingness to pay of €18,000. Varying monetary threshold values up to €80,000 showed a similar pattern. Findings suggest that the video-based intervention was far more cost-effective than the text-based intervention and the brief generic advice that respondents received in the control condition, since it resulted in more quitters and lower societal costs. The cost-utility analyses with quality of life as outcome measure revealed a different pattern. The QALYs gained by the video condition during the one year follow-up period were almost the same as those gained among respondents in the control condition, with far greater costs in the video-based CT intervention. Consequently, in terms of QALYs, the control condition seemed to be the most preferable treatment when using a monetary threshold of €0 to €18,000. When increasing the threshold value for an additional QALY up to €80,000, the results changed and showed a preference for the video condition. The results from sensitivity analyses revealed comparable results. When cost estimations varied, the video-based CT intervention remained the most cost-effective treatment, whereas cost-utility was still the highest within the control condition.

Consistent with findings from other economic evaluations of smoking cessation interventions [19], [20], [23], our results indicated that the video-based CT smoking cessation intervention is likely to be most cost-effective. Compared to the control condition, €1,500 had to be paid within the video-based intervention per additional 1% probability of abstinence. This amount is somewhat lower than what was found in previous research [23], [48]. Nevertheless, these results are difficult to interpret since little information is available on the amount of money that society is willing to pay for smoking cessation and as a result no accepted cut-off points exist for smoking abstinence rates [23].

Since difficulties may arise when policy makers try to compare results from cost-effectiveness analyses in which the cost-effectiveness ratios are expressed as concerning different outcome measures, we furthermore included the cost-utility analyses to our study. With regard to cost-utilities, if society is willing to pay only a small amount per QALY gained, our results showed that the control group might be the most preferable treatment compared to both CT smoking cessation interventions. Comparable results were found in a previous study [23], where the control condition (usual smoking cessation care in the general practice setting) was also more preferable compared to a CT smoking cessation intervention and a CT smoking cessation intervention with face-to-face counseling by a practice nurse. One possible reason for this finding might be that the follow-up period of twelve months was not long enough to observe improvements in quality of life. Previous economic evaluations in respondents with smoking-related diseases indicated that quality of life could only be detected with longer time periods [49], [50]. One possible explanation for the different results of the cost-effectiveness and cost-utility analyses could be that respondents may not have perceived improvements in their quality of life during the one-year follow-up period used in the present study. Since we used a relatively short follow-up period of twelve months, respondents may not have perceived any direct health benefits in the short term, due to possible withdrawal symptoms associated with quitting [51], [52]. Although other studies did identify a positive association between smoking abstinence and quality of life yet over a twelve-month follow-up period [53], [54], future economic evaluations should include longer time horizons in order to detect an improved quality of life for respondents who quit smoking.

### Strengths and limitations

To our current knowledge, this was one of the first studies examining the cost-effectiveness and cost-utility of a video-and text-based CT intervention for smoking cessation. In order to compare the three different conditions more specifically, the effects of the current study were not only assessed in terms of quality of life but also in terms of smoking abstinence. The inclusion of this cost-effectiveness analysis (using smoking abstinence as outcome measure) allows policy makers to compare the net benefit of the intervention to other smoking cessation interventions. Moreover, several sensitivity analyses were conducted to test for uncertainty of parameter estimates which can be seen as another strength of our study.

Yet, several limitations of the current study should also be discussed. First, we interpreted our results at varying monetary threshold values of smoking abstinence and QALY up to €80,000. However, until now no information has been available on the exact amount of money that society is willing to pay for smoking cessation, i.e. per additional ex-smoker, which makes interpretation difficult. Although the WTP of €18,000 is an accepted Dutch-cutoff point for measuring the QALY [55], past research has suggested varying these WTP values [56]. Nevertheless, we assume that the chosen range of our study from €0 to €80,000 was broad enough to capture all important threshold values. As already recommended by previous research [23], future studies should however aim to determine a cut-off point for the WTP per abstinent respondent in order to enable an accurate interpretation of the findings from the present and similar studies. Second, we were not able to detect improvements in quality of life between the different conditions. For future economic evaluations regarding smoking cessation interventions, it may be useful to include other operationalizations of the QALY or other outcomes, which may be better able to detect short-term health changes (e.g. non-health-care-related outcomes such as satisfaction or enablement) [57], [58]. Third, health-care utilization was based on self-reported data, which might have introduced recall bias. However, we tried to keep recall bias low by using a three-month instead of six-month retrospective questionnaire [59], [60]. Upcoming economic evaluation studies may, however, want to include more objective measurements (such as data from health insurance, or medication registration from the pharmacy or medical professions) of health-care utilization. At last, although attrition rates in our study were relatively low compared to dropout rates in other Internet-based smoking cessation intervention trials [2], [5], [8], we replaced missing data using several imputation techniques that are commonly used to impute missing data in cost-effectiveness studies [23], [40] and are recommended in the smoking cessation literature [43]. Yet, the most optimal strategy for missing data imputation is currently not evident and it may thus be that our strategy could have influenced the results presented. Missing values on the primary outcomes measure were replaced using a negative approach, in which respondents lost to follow up were regarded as smokers. Although this approach is widely used, it might have been too strict since many dropouts might actually have been quitters no longer in need of the intervention, rather than smokers. Therefore, this approach might have resulted in an underestimation of the intervention's effectiveness [61], [62], and possibly its cost-effectiveness. As web-based interventions have been found to be subject to relatively large attrition rates, future research is needed to estimate the most optimal strategy for handling missing data in cost-effectiveness trials that evaluate web-based smoking cessation interventions.

## Conclusions

The results of our study revealed that the video-based CT smoking cessation intervention had the highest probability of being cost-effective. The video-based intervention was far more cost-effective than the text-based intervention and the brief generic advice in the control condition, since it resulted in more quitters and lower societal costs. Yet, the cost-utility outcomes tended to be in favour of the control condition, but interpretation of this finding is hindered by the relatively short follow-up and great likelihood of insensitivity of the QALY assessment. Future studies need to assess which QALY measure may be the most sensible method to detect short-term improvements regarding quality of life. Furthermore, more research should concentrate on the most optimal strategy for handling missing data in economic evaluations of web-based smoking cessation interventions. Finally, more research is needed to identify an acceptable cutoff-point for the WTP per abstinent respondent in order to interpret incremental costs in future studies concerning the cost-effectiveness of smoking cessation interventions.

## Supporting Information

Checklist S1 **CONSORT checklist.**
(DOC)

Protocol S1 **Trial protocol.**
(PDF)
