# Supplementary material for: An Economic Evaluation of a Video- and Text-Based Computer-Tailored Intervention for Smoking Cessation: A Cost-Effectiveness and Cost-Utility Analysis of a Randomized Controlled Trial
Source: PLoS One. 2014 Oct 13;9(10):e110117. doi: 10.1371/journal.pone.0110117 (PMC4195710; doi:10.1371/journal.pone.0110117)
Supplement: Protocol S1 — Trial protocol. (PDF) [file pone.0110117.s002.pdf]

Stanczyk et al. BMC Public Health 2011, 11:847
http://www.biomedcentral.com/1471-2458/11/847

BMC Public Health

STUDY PROTOCOL Open Access

Study protocol of a Dutch smoking cessation e-health program

Nicola E Stanczyk[1,2*], Catherine Bolman[1,3†], Jean WM Muris[1,4†] and Hein de Vries[1,2†]

Abstract

Background: The study aims to test the differential effects of a web-based text and a web-based video-driven computer-tailored approach for lower socio-economic status (LSES) and higher socio-economic status (HSES) smokers which incorporate multiple computer-tailored feedback moments. The two programs differ only in the mode of delivery (video- versus text-based messages). The paper aims to describe the development and design of the two computer-tailored programs.

Methods/design: Respondents who smoked at the time of the study inclusion, who were motivated to quit within the following six months and who were aged 18 or older were included in the program. The study is a randomized control trial with a 2 (video/text) * 2(LSES/HSES) design. Respondents were assigned either to one of the intervention groups (text versus video tailored feedback) or to the control group (non-tailored generic advice). In all three conditions participants were asked to fill in the baseline questionnaire based on the I-Change model. The questionnaire assessed socio-demographics, attitude towards smoking, knowledge, self-efficacy, social influence, depression, level of addiction, action planning, goal actions, intention to quit smoking, seven-day point prevalence and continued abstinence. Follow-up measurements were conducted at six and twelve months after baseline.

Discussion: The present paper describes the development of the two computer-tailored smoking cessation programs, their components and the design of the study. The study results reveal different working mechanisms of multiple tailored smoking cessation interventions and will help us to gain more insight into effective strategies to target different subgroups, especially smokers with a lower socio-economic status.

Trial registration: Dutch Trial Register NTR3102

Background

Smoking tobacco is one of the most preventable causes of illness and premature death in the world [1]. The development of effective smoking cessation programs is essential to prevent illnesses [2].

One effective strategy for health promotion that has been developed during the last decades concerns computer-tailored interventions [3,4]. CT has developed since the 1990s as a new technique for health promotion, as it provides the individual with personalized information and feedback on health behaviour. Tailored health messages are based on knowledge of a person generated from his or her answers to a questionnaire on issues related to health behaviour (in the case of smoking, for example, the perceived advantages of smoking and perceived support to quit smoking). Research has demonstrated that tailored communication attracts and keeps the individual's attention [5]. Tailored information has furthermore been shown to be more likely to be read, remembered and considered personally relevant [6]. CT programs can be either print delivered (delivered by post) or web-based. One advantage of web-based CT is that the user can follow the program in privacy at any preferred time [7]. Furthermore, multimedia components can be incorporated and it has the potential to reach large audiences. CT is therefore increasingly offered by the web.

* Correspondence: nicola.stanczyk@maastrichtuniversity.nl
† Contributed equally
[1]Department of Health Promotion, School for Public Health and Primary Care (CAPHRI), Maastricht University, P.O. Box, 6200 MD Maastricht, the Netherlands
Full list of author information is available at the end of the article

© 2011 Stanczyk et al; licensee BioMed Central Ltd. This is an Open Access article distributed under the terms of the Creative Commons Attribution License (http://creativecommons.org/licenses/by/2.0), which permits unrestricted use, distribution, and reproduction in any medium, provided the original work is properly cited.

In the Netherlands, a range of empirical studies has demonstrated the efficacy of web-based computer-tailored programs compared with non-tailored programs for several lifestyle behaviours [6,8] including smoking cessation [8-11]. The effectiveness of computer-tailored technology to smoking cessation has been reviewed and tested in various studies [12,13].

Smoking prevalence in Dutch people with a low socio-economic status (LSES) is higher than in people with a high socio-economic status (HSES) [14]. People with a low SES also begin to smoke at an earlier age [15] and have more difficulties in quitting [16]. Several studies suggest that health communication probably needs to be different for LSES populations and suggest an approach with less cognitive effort [17-19] that focuses more on visual than on text information [20].

Consequently, the potential problem with CT smoking cessation programs is that they rely heavily on text-based messages and might therefore be less attractive to LSES smokers. Cognitive psychologists emphasize learning from more than one modality source, since humans actively process information with separate systems for visual and verbal representations [21]. Several studies suggest that videos may be more effective in attracting attention and stimulating comprehension, especially in LSES smokers and smokers unmotivated to change [22]. Video messages have been shown to require less mental effort and less translation of abstract concepts to create images [23]. Findings using the Elaboration Likelihood Model suggest that LSES groups often do not process information deeply [20]. Furthermore, research has identified that video presentation activates both visual and verbal channels, which together may lead to better learning outcomes [21]. Whereas text messages require translation of abstract ideas to concrete situations, video messages may help to focus the recipient more on the basic message and help their understanding of the core message and reasons for engaging in the desired behaviour. Lower educated and motivated groups may thus profit in various ways from the utilization of videos and illustrations in smoking cessation programs.

To test the effectiveness of video-based messages it is important to use an experimental design in which the information provided by the videos is comparable to the text messages and a control condition. The video information should therefore use the same basic messages as the text version [24]. Yet as far as we know no studies have been executed which test whether the use of video-based messages alone (without any other animation effects, like cartoons, hyperlinks, etc.) has an added effect on smoking cessation especially when targeting less literate groups. Also little attention has been given yet to the possible efficacy of video-based computer-tailored smoking cessation interventions targeting LSES smokers.

Besides method strategy it is also important to consider the dosage of CT. Research has demonstrated that multiple smoking cessation CT results in greater cessation effects than single tailored feedback letters [9]. Similar effects have also been found for other behaviours than smoking, e.g. physical activity and dietary behaviour [7,25]. Multiple tailoring feedback moments are therefore considered to be a useful strategy to implement for web-based computer-tailored smoking cessation programs as well.

The study whose protocol is described here aims to include these different elements by developing and testing two new e-health programs, incorporating multiple computer-tailored feedback moments. The two e-health programs differed only in the mode of delivery that was used (video- versus text-based messages).

The main aim of this paper is to describe the development of the two computer-tailored smoking cessation programs, their components and the design of the efficacy study.

Methods/design

The study protocol was submitted for approval to the Medical Research Ethics Committee (MREC) of Atrium Medical Centre Heerlen. The MREC reviewed the research protocol and judged that no further MREC approval was necessary for this study because patients were not obliged to a certain act. Furthermore, the questionnaires of the intervention were judged not to have a deep psychological impact. Human subjects' approval was obtained in line with the APA informed consent ethical principles. At the beginning of the study, all eligible participants were provided with information on the study and asked to sign informed consent forms.

Target population and Inclusion criteria

Only respondents who smoked at the time of the study inclusion, who were motivated to quit within the following six months and who were aged 18 or older were included in the study. Furthermore, respondents needed to have internet access and had to be able to understand the Dutch language sufficiently.

Study design

Respondents were randomly assigned to one of the two experimental conditions or the control condition.

1. Text computer-tailoring: respondents received computer-tailored text messages during several feedback sessions. The number of feedback sessions was dependent on the respondents' intention to quit

smoking and their readiness to set a quit date within a month.

2. Video computer-tailoring: respondents received computer-tailored video messages during several feedback sessions. The number of feedback sessions was dependent on the respondents' intention to quit smoking and their readiness to set a quit date within a month (the text messages mentioned in point 1 were replaced by videos).

3. Control group: participants received one short piece of generic text advice.

More detailed information is provided in the 'intervention' section.

Procedure

Recruitment

Participants were recruited by several recruitment strategies. First, smokers were recruited through general practitioner (GP) practices. We asked about 150 GP practices to refer smoking patients to our intervention website. The GP practices received a letter which entailed a description of the project, tasks for the GP and the assistant and all necessary recruitment materials. GPs willing to participate in our study were asked to refer a minimum of 20 patients who met the inclusion criteria to the intervention website over a period of 12 months.

Second, respondents were recruited by a mass media approach, which consisted of calls in local newspapers and websites of national health funds (the Dutch Diabetes foundation, the Dutch Cancer Society, the Dutch Foundation for a Smoke-free Future and the Dutch Asthma Foundation). The calls directly referred smokers who were interested in participating in the study to the intervention website where they could find more information about the project and registration.

Third, smokers were also recruited via several Dutch companies. We asked companies to bring the program to the attention of their employees by means of advertisements, announcements in staff magazines, internal websites and/or by any other channels they used to communicate with their employees. Similarly to the other recruitment strategies, calls referred employees to the intervention website.

The intervention website gave information about the content of the program and explained that three different versions of the program were to be tested. Respondents were informed that they would be randomly allocated to one of the three different program versions. Participants were told that they could win 100 € after completing all parts of the program and the follow-up measurements at six and twelve months.

It was decided to use these mixed recruitment strategies because previous studies by the Department of Health Promotion at Maastricht University showed these strategies are sufficient to reach many smokers who are representative of the Dutch population of smokers and could also help to reach a sufficient number of LSES smokers [8,26].

Randomization

Smokers interested in quitting within the following six months were invited to visit the intervention website and could sign up with their own username and password. After signing up for participation, giving online informed consent and passing through the inclusion criteria, respondents were allocated to one of the three conditions of the program. Randomization was performed automatically by Tailor Builder computer software (OSE, Sittard, the Netherlands). This software was especially developed for the execution of web-based computer-tailored programs [27].

Study course

The course of the study is described briefly below. The main elements of the intervention are outlined in the Materials section.

The Tailor Builder software randomly assigned respondents either to one of the intervention groups (text vs. video-tailored feedback) or to the control group (non-tailored generic advice). Respondents were not informed of the group to which they were allocated (see also Figure 1). The video-based condition used the same computer-tailored messages as the text-based condition. Respondents were asked to answer three questions concerning the inclusion criteria (18 years or older, smoker, intention to quit within a year or earlier). Respondents who did not meet the inclusion criteria were subsequently excluded from the study and received a message explaining why they could not participate in the study. Respondents who did not meet the inclusion criteria were subsequently excluded from the study and received a message explaining why they could not participate in the study.

All other respondents were assigned to one of the three conditions, continued with the program and were asked to fill out the baseline questionnaire. At the end of the baseline questionnaire, respondents were asked to fill in whether they had plans to quit within the following month. Depending on their answer, respondents were allocated to one of two possible routings of the program.

Respondents with the intention to quit within the following month followed route 1 (detailed description of route 1, see Materials section). Respondents were asked to choose a quit date and were invited seven days before their quit date to participate in the second session of the program. Respondents subsequently received an

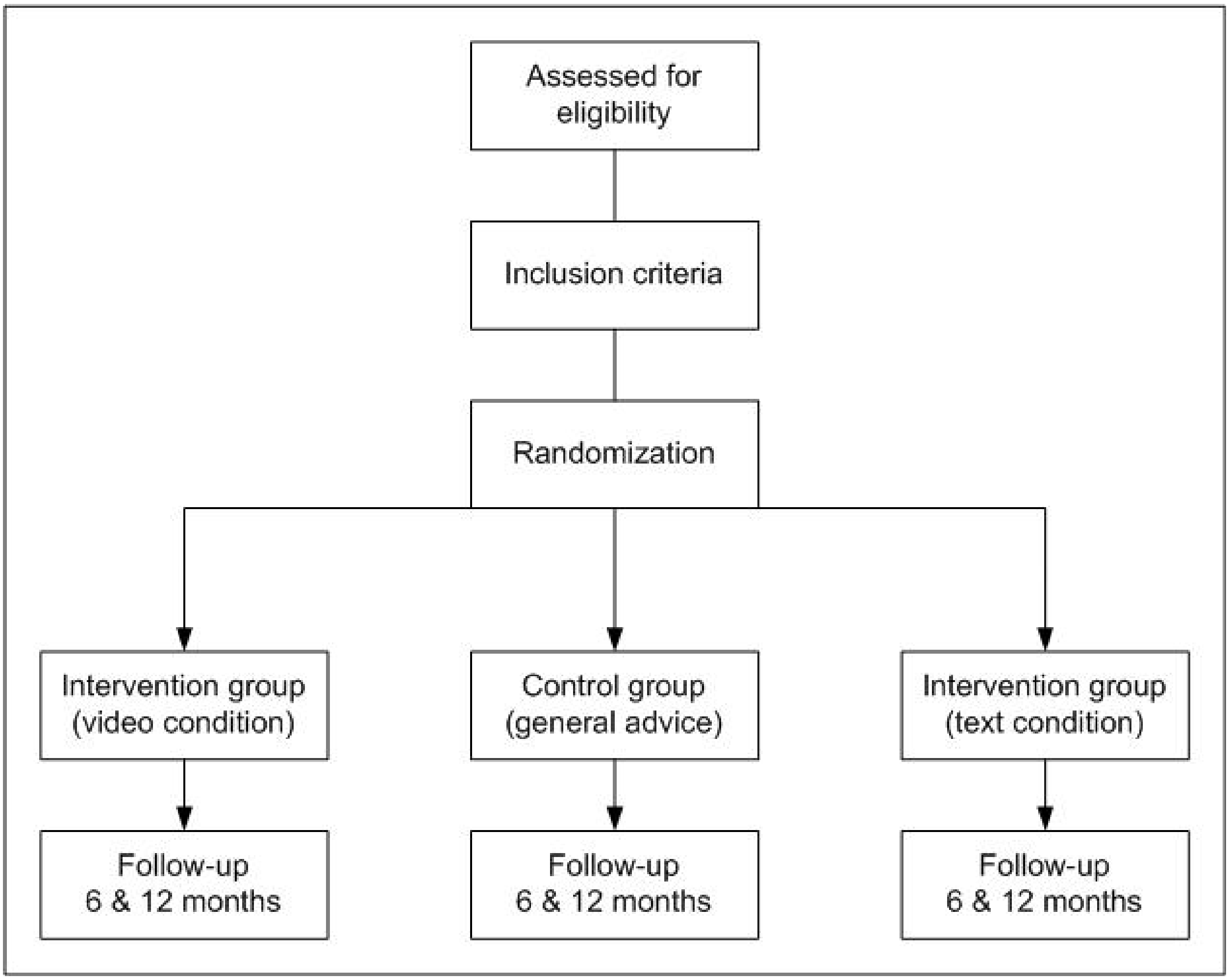

Figure 1 Flowchart of the randomized control trial.

invitation for the third session three days after their quit date. Additionally, respondents received an invitation for the fourth, fifth and sixth feedback sessions two, four and eight weeks after their chosen quit-date.

Respondents not intending to quit within the next month followed route 2 (for details see Figure 2). One month after the baseline questionnaire respondents were invited by the program to follow the second session. At session two they were asked again to indicate their intention to quit within the following month. Respondents prepared to quit were subsequently directed to route 1, as described above. Respondents not prepared to quit received their last invitation for the third session one month later.

Six and twelve months after filling out the baseline questionnaire, all respondents of the three conditions were asked to complete the follow-up measurements.

In the e-health programs, invitations for the feedback sessions were sent by e-mail. Next, respondents received a personalized summary report by e-mail after each feedback session. Respondents in the text condition had the opportunity to reread and print the tailored text messages. Respondents in the video condition were able to watch the tailored video messages again. Respondents in the control condition were not able to reread the short piece of generic text advice. The average time of the different intervention sessions was about 20 minutes.

Materials

Questionnaires

In all three conditions participants were asked to fill in the baseline questionnaire.

The baseline questionnaire was based on the I-Change model [3] and has previously been used in other studies that assessed the impact of computer-tailoring on life-style changes, like smoking [8,26]. The baseline questionnaire consisted of questions on smoking behaviour, smoking-related beliefs, social influence, self-efficacy to

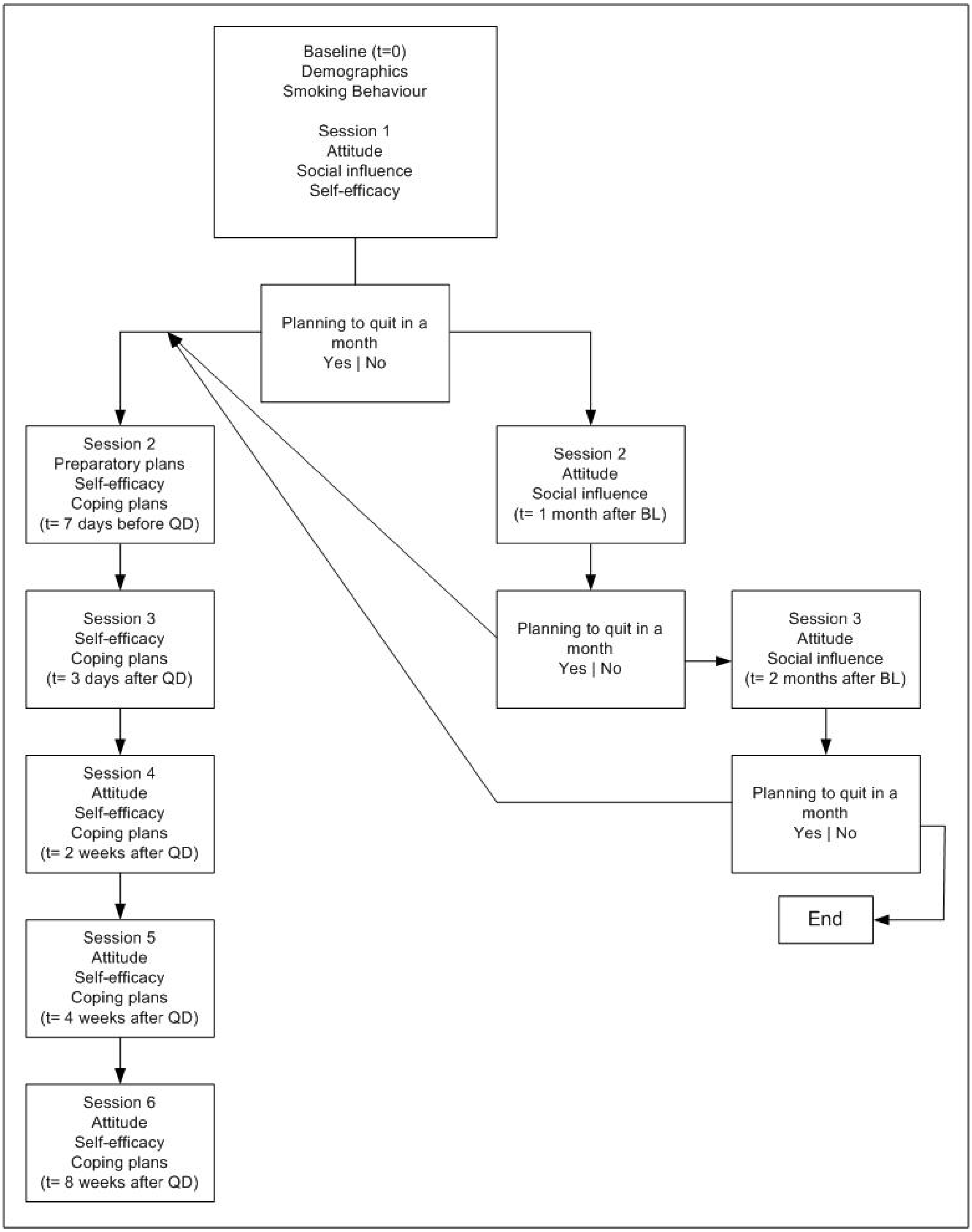

Figure 2 Flowchart of the intervention.

quit, preparatory planning and stage of change [8,28]. Furthermore, existing scales were used to measure cognitive processing (Elfeddali I, Bolman C, Candel MJJM, Wiers RW, De Vries H: Preventing smoking relapse via Internet-based computer tailored feedback: 12 month results of the SQ4U-study, submitted) and depressive complaints [29]. The follow-up questionnaires at six and twelve months assessed smoking behaviour [28], stage of

change [8], quality of life (QoL) and healthcare costs [30].

Social demographic information

Variables assessed were: age, gender, marital status, religious background, ethnic background, education level, current work status, income level.

Health status

Occurrence of smoking-related diseases was measured by four questions on a dichotomous scale (Do you suffer from chronic respiratory disease, cancer, diabetes or cardiovascular disease?; 0 = no; 1 = yes; index: 0 = no disease to 4 = occurrence of four diseases) [8].

Level of depression

Level of depression was measured by the abbreviated CES-D by four items (e.g. During the past week I felt depressed; 1 = rarely or none of the time (less than 1 day); 2 = some or a little of the time (1-2 days); 3 = occasionally or a moderate amount of time (3-4 days); 4 = most or all of the time (5-7 days) [31].

Cognitive processing

Cognitive processing was measured by six items on a five-point scale of the Heuristic Systematic Processing Questionnaire (e.g. I like tasks where I do not have to think much; 1 = I totally disagree; 2 = I disagree; 3 = I do not know; 4 = I agree; 5 = I totally agree) [32].

Media preference style

Media preference style was assessed by a six-item questionnaire. The questionnaire which was developed by the research team assessed whether respondents preferred the visual or auditory channel in order to understand and remember new information (e.g. I can understand new information better if 1 = I can read it; 2 = the information is reported by a person in a film; I remember information better if 1 = I can read it; 2 = the information is reported by a person in a film).

Level of addiction

Level of addiction was measured by six items, asking respondents how many cigarettes/shags they smoked per day, at which time points and whether they had difficulties not smoking in smoke-free places. The answers to these six questions were converted into an overall score, ranging from 0-10 [33].

Smoking behavior

Smoking behaviour was measured by asking respondents how many cigarettes/shags they smoked per day. Smoking behaviour was converted into an overall score (number of cigarettes a day) [25].

Habit

Smoking habit was assessed by an abbreviated version of the Verplanken and Orbell's Self-Reported Habit Index of six items with which respondents could agree or disagree on a five-point scale (e.g. Smoking is something which I do automatically; 1 = I totally disagree; 2 = I disagree; 3 = I do not know; 4 = I agree; 5 = I totally agree) [34].

Attitude

Attitude towards the rational and emotional pros and cons of smoking was measured by twelve items with which respondents could agree or disagree on a five-point scale (e.g. When I do not smoke, my condition improves; 1 = I totally disagree; 2 = I disagree; 3 = I do not know; 4 = I agree; 5 = I totally agree) [8,25,35].

Social influence

Social influence was measured by two existing scales; a social support scale and a social modelling scale [25]. Two items measured whether people in the respondents' environment smoked (respectively partners and people in the direct environment (Does your partner smoke?; 0 = no;1 = yes, 9 = not applicable; How many of the people in your environment smoke?; 1 = none of them; 2 = the minority; 3 = half of them; 4 = the majority; 5 = all of them; 9 = not applicable). The second scale assessed whether the person received social support in favour of quitting or against quitting on a five-point scale (My partner/people in my environment; 1 = do not support me; 2 = support me a bit; 3 = support me; 4 = support me a lot; 9 = not applicable) [25].

Self-efficacy

Self-efficacy was measured by nine items on a five point scale (e.g. Do you think you will manage not to smoke when you drink a cup of coffee?; 1 = definitely not; 2 = probably not; 3 = maybe yes; 4 = probably yes; 5 = definitely yes) [36].

Action plans

Action plans were assessed by five items. Participants had to indicate on a five-point scale whether they planned to execute different preparatory plans for their quit attempt (e.g. removing ashtrays, asking guests not to smoke and thinking about difficult situations they might encounter after quitting; 1 = surely not - 5 = surely yes) [25].

Coping plans

Coping plans were assessed by nine items. Respondents had to indicate whether they had made any plans to prevent relapse in difficult situations (e.g. plans how to cope with withdrawal symptoms, how to cope with negative mood and how to cope with high risk situation such as e.g. parties or being with friends; 0 = no; 1 = yes) [37].

Readiness to quit smoking

Readiness to quit smoking was measured by one item assessing whether the respondent intended to quit smoking on a six-item scale (1 = yes, within the following month; 2 = yes, within one and three months; 3 = yes, within four and six months; 4 = yes, within one year; 5 = yes, within one and five years; 6 = yes, but not within the following five years) [25,35].

Intervention

Intervention elements of the two e-health programs

The theoretical rationale behind computer-tailoring is to make the information that the user receives as personally relevant as possible. According to theories of information processing, e.g. the Elaboration Likelihood Model, people pay more attention to personally relevant information, thoughtfully consider it and appreciate it more than non-personally relevant information [25]. Research has furthermore shown that information which is attended to and thoughtfully considered is more likely to influence a person's beliefs and behaviours [5].

The tailored program consisted of a screening instrument for smoking behaviour, and a feedback library with all pre-written feedback messages and tailoring algorithms. The tailoring algorithms linked up the specific answers of the respondent with the relevant corresponding health message in the feedback library. The screening instrument consisted of different questionnaires, which were developed to assess the smoking behaviour itself, the perception of the smoking behaviour and determinants such as attitude, social norms and self-efficacy towards smoking. After completion of the questionnaires, automatized feedback was generated and provided based on the specific answers the respondent gave. Feedback was provided on item level (e.g. for each coping plan).

The video-based condition used exactly the same tailored messages as the text-based condition. The video had a new reading format with five different adults delivering the messages (two males, three females) who were chosen as a result of screen tests. In the context of learning, research has shown that students learn significantly more and are more motivated when information is presented through multiple visual agents of different gender and race. This can be explained by the fact that different information might be better understood and separated if it is delivered by separate agents [38]. With regard to the selection of the speakers we therefore used a mix of adults in terms of age, gender and ethnicity. Research has also shown that individuals are more influenced by visual agents who look similar to themselves regarding appearance- related characteristics [39]. We therefore tried to choose normal adults with normal everyday clothes with which smokers can easily identify. We furthermore aimed to exclude adults with strong dialects in order to avoid distraction or lack of comprehension.

Respondents in the two experimental conditions received personalized information in several feedback sessions, depending on a smoker's readiness to plan a quit date within the following four weeks (see previously described study course). The feedback messages of the program aimed to increase the respondents' positive attitude toward quitting, to increase the respondents' motivation to make a quit attempt and to support the respondents during the period after their quit attempt. Multiple feedback moments were included in the program because they have been shown to be more effective in encouraging health behaviour than a single feedback moment [6].

Baseline computer-tailored session

The baseline computer-tailored session consisted of a shortened version of a previously tested CT program that was found to be more effective in increasing smoking cessation than the provision of generic advice and information [8,25]. The baseline session was intended to increase the respondents' motivation to quit smoking and to encourage the respondents to set a quit date in the next few months. Respondents received tailored feedback on the perceived advantages and disadvantages of quitting. Tailored feedback was offered with regard to the different pros of quitting (e.g. decreased likelihood of diseases) in order to convince the respondents of the advantages of quitting. Tailored feedback further addressed the disadvantages of quitting (e.g. withdrawal symptoms) in order to combat possible negative existing beliefs. With respect to the respondent's perceived social support, tailored feedback dealt with how to deal with smokers in their environment (e.g. asking them not to smoke in their environment in order to avoid a relapse). Furthermore, respondents received feedback on their perceived self-efficacy, the importance of self-efficacy for successful quitting and tips for increasing self-efficacy. Lastly, information was provided on how to plan quitting (e.g. planning a quit date and using smoking cessation aids). Depending on the readiness to quit within one month or not, respondents received feedback in one of the two routings that are explained in detail below.

Routing 1

Respondents who were ready to quit in the next month were directed into routing 1. After the baseline questionnaire, respondents in session 1 were asked to set a quit date. Respondents were requested to choose a quit date between eight days and one month from baseline. At the end of the first session, smokers were informed by e-mail that they would be invited for the next session of the program one week before their quit date in order to receive new support and help in preparing their quit attempt in the following days.

One week before their quit date respondents were invited by e-mail to follow the next session of the program. The second session was aimed at preparing the smoker to quit successfully and to be prepared for the difficulties that might arise after quitting. Respondents were first asked to fill out a short questionnaire on self-efficacy and action planning items. Several studies identified a low level of self-efficacy as a good predictor of

smoking relapse (Elfeddali I, Bolman C, Candel MJJM, Wiers RW, De Vries H: Preventing smoking relapse via Internet-based computer tailored feedback: 12 month results of the SQ4U-study, submitted) [40-42]. Therefore, respondents received feedback on how to increase self-efficacy. Tailored feedback on self-efficacy was provided in order to help the respondent to gain confidence to quit and to help him/her to deal with the possible difficulties and negative consequences of their quit attempt. Next, respondents were encouraged to make preparatory plans for their quit attempt. Tailored feedback addressed the advantages of making plans in advance and respondents were furthermore stimulated to think about concrete plans (e.g. removing ashtrays, asking guests not to smoke and thinking about difficult situations they might encounter after quitting). Lack of preparatory planning has been shown to be a good predictor of smoking relapse and preparing for quitting has been shown to increase smoking cessation [8]. The second session not only aimed at preparing the smoker to quit successfully (by encouraging preparatory planning) but also to prepare the smoker to cope with the challenges/difficulties that might arise after quitting. This so-called coping planning has been shown to be a helpful strategy for dealing with high-risk situations [43]. To encourage coping planning respondents were first asked to choose from a list of eleven risk situations (e.g. I find it difficult not to smoke if I am angry, I find it difficult not to smoke when I see another person enjoying a cigarette) the three most personally relevant high-risk situations. Next, respondents were asked to indicate whether they had already thought about how to deal with these high-risk situations. If they had not thought about coping plans, respondents were instructed to formulate specific coping plans for their personal high-risk situations. Examples were given of how to make concrete plans (e.g. Next time you feel angry, instead of smoking a cigarette, go outside and have a walk).

Session 3 took place three days after the quit date of the respondent. At that time, respondents were invited by e-mail to turn to the CT program again and to fill out a brief questionnaire on self-efficacy and coping planning items. The third session was aimed at encouraging the respondent again to think about possible coping plans in order to deal with high-risk smoking-related situations. The same strategy was used as in session 2. Respondents were also asked about their smoking behaviour since their quit date. In cases of lapse/relapse the respondent was told that smoking cessation is a process in which lapses may occur and that they can be used positively in continuing the quit attempt. Respondents were also encouraged and invited to continue their quit attempt or to plan a new quit date. This strategy derived from a CT relapse prevention preprogram that has shown to be effective in preventing relapse (Elfeddali I, Bolman C, Candel MJJM, Wiers RW, De Vries H: Preventing smoking relapse via Internet-based computer tailored feedback: 12 month results of the SQ4U-study, submitted).

Two weeks after the quit date respondents were invited for session 4. Respondents were instructed to fill out again a brief questionnaire on self-efficacy and coping planning items. The same procedure was used as in session 2 and 3 of the program. Furthermore, pros and cons of quitting were assessed again as in session 1. This was done in order to encourage the respondents to rethink about the pros and cons of smoking and quitting that they perceived and to assess whether respondents had acquired possible new pros of quitting since the first session of the program.

In sessions 5 and 6, after four and eight weeks, a similar strategy was used to that in session 4. During the last three sessions, respondents could still choose to receive tailored feedback on different items (such as how to cope with negative moods or how to cope with high-risk situations). This option was provided since we expected them to encounter different problems throughout their quit attempt. In all these sessions smokers who had reverted to smoking were invited and encouraged to restart their quit attempt.

Routing 2

Respondents who were not ready to quit in the next month followed routing 2. At the end of session 1, that followed immediately after the baseline session, respondents were encouraged to use the next month to reflect on their quitting intention and the tailored information they received during session 1. After one month these smokers received an e-mail with an invitation for the second session. In session 2 the respondent was asked about his/her smoking behaviour. Subsequently respondents again received feedback on the perceived advantages and disadvantages of smoking and quitting and how best to obtain social support from their environment. At the end of the session smokers were asked to indicate their readiness to quit in the next month. Respondents who were ready to quit in the next month and ready to plan a quit date were sent to routing 1. Respondents not intending to quit in the next month were informed that they would receive an invitation for session 3 one month later to reassess their smoking behaviour and their motivation to quit.

As in session 2, respondents received in session 3 tailored feedback on the perceived advantages and disadvantages of smoking and quitting and how best to obtain social support from others. Smokers were again encouraged to quit and were asked at the end of the session to indicate their intention to quit in the next month. Respondents ready to quit within the next

month and ready to plan a quit date were sent to routing 1. Smokers not prepared to plan a quit date within a month received an empathic message, indicating that it was respected that they were not ready to quit smoking and that we would therefore send no further e-mails at this stage.

Finally, at the end of the program, all respondents were informed that they would receive an invitation (by e-mail) after six and twelve months to fill out a brief follow-up questionnaire about their smoking behaviour and a process evaluation questionnaire on the programs after 6 months.

Control group

The control group received brief generic text advice about smoking cessation. The content of the generic text was similar to the experimental conditions. Although brief advice was given on how to quit successfully, the generic text advice was not tailored to personal factors and respondents in the intervention group all received the same information. Furthermore, respondents were not provided by e-mail with an overview of the brief non-tailored text advice.

Outcomes and biochemical validation

Primary outcome measurement

Primary outcome measures of this study measured at six and twelve months after baseline were seven-day point prevalence abstinence from smoking (PPA), continued abstinence and prolonged abstinence. Seven-day point prevalence was defined as not having smoked during the last seven days (measured from follow-up). Continued abstinence was defined as not having smoked since the last quit date, whereas prolonged abstinence took into account a grace period of two weeks [28,44]. During this grace period re-initiating of smoking behaviour after the quit date was not defined as a relapse. All outcome variables were measured according to the definitions in Hughes et al. [28,44]. Smoking during the last seven days and after the personal quit date was coded as relapse (0), whereas non-smoking during the last seven days and since the quit date was coded as abstinence (1).

Secondary outcome measures

Secondary outcome variables measured at six and twelve months after baseline included 24-hour point prevalence abstinence (having smoked during the last 24 hours; 1 = no; 0 = yes), having made a serious quit attempt (not having smoked for at least 24 hours; 1 = no; 0 = yes) and changes in smoking behaviour since baseline measurements [44]. All respondents were further asked to fill out a process evaluation questionnaire at the six-month follow-up to evaluate their experience of the program.

Biochemical validation

At the last follow-up measurement (12 months after baseline) self-reports regarding cessation will be biochemically validated by means of a cotinine test. We aim to validate self-reports randomly in a subsample of participants who have indicated abstention from smoking. At least 50% of this sample will be invited to participate. We aim to conduct the biochemical validation within one week after 12-month follow-up measurement. Respondents reporting abstention at the 12-month follow-up will be approached by a research assistant to make an appointment for the cotinine test (e.g. at home or at work). Saliva of the respondents will be collected with a swab stick and will be applied to a test strip. Respondents who decline to undergo the test will be asked by the research assistant for the reason(s) why.

Statistical analysis

Sample size calculation

Sample size calculations were based on the ability to detect 10% differences between the three conditions with a power of 0.80 and an alpha of 5%. To calculate the sample size, seven-day point prevalence abstinence at the six- and twelve-month follow-up was taken as the base since it is considered to be the most sensitive and valid measure of smoking cessation [45].

Power calculations were made separately for LSES and HSES groups. For the LSES group we expected that the video condition will result in an 18% quit rate, and the text condition in an 8% quit rate, requiring 176 participants in both conditions. For the HSES group we expected quit rates of 22% in the text-based condition and 12% in the video-based condition, requiring 220 smokers for both conditions.

Attrition prevention

Different strategies have been applied in order to prevent high attrition rates. First, respondents completing all questionnaires are eligible to win a price of 100€. Second, the follow-up questionnaires at six and twelve months are brief, only aiming to assess behaviour, readiness to quit smoking, since this strategy has been found to increase participation rates by approximately 8 to 10%. Participants who do not respond to the follow-up measurement at six and twelve months receive two reminders inviting them again to fill in the follow-up measurements. Respondents who still do not react will receive an invitation by e-mail to briefly indicate their current smoking status by indicating whether they have smoked during the last 24 hours, in the last week or in the last months, since this strategy was shown in another study to result in an additional response rate of 8 to10%.

Analysis

We will conduct logistic regression analysis at six and twelve months to assess program effects on the main outcome variables. We will further perform linear regression analysis and covariance analysis to assess effects on secondary outcome variables at six and twelve months. We will correct for baseline factors (e.g. demographics, smoking behaviour, attitude, intention) in both types of regression analyses by adding these variables as possible confounders. Interaction effects of experimental condition (text vs. video) and SES will be explored by moderation in the logistic regression analysis since we expect an interaction effect between SES and condition.

Additional studies

Additional to the effect study we will conduct two other studies in the same trial, a process evaluation and an economic evaluation, which are described briefly below.

Process evaluation

We will conduct a process evaluation to assess the respondents' reactions to the program. Furthermore we will assess differences in evaluation between LSES and HSES smokers. The questionnaire of the process evaluation is based on previously used questionnaires [6,25] and assesses information appraisal of the tailored advice. The concepts attention, comprehension and appreciation of the tailored advice are measured on a five-point scale. Attention is measured by assessing whether the respondents felt that the messages attracted their attention and retained it. Comprehension is measured by asking whether they understood the messages and whether they found them difficult or encountered difficulties with words or passages. Appreciation of the advice is measured by asking whether the respondents liked the messages and whether they found the message personally relevant.

Economic evaluation

The economic evaluation involves a combination of a cost-effectiveness analysis (CEA) and a cost-utility analysis (CUA) to analyse whether the e-health program is preferable in terms of cost, effects and utilities from a societal perspective. In the CEA, the incremental cost effectiveness ratio (ICER) is expressed as the incremental costs per additional quitter (measured as 12 months' Point Prevalence Abstinence). In the CUA, the outcome measure is quality-adjusted life years (QALYs), which is based on the EuroQol utility score [30].

Discussion

The paper describes two multiple computer-tailored smoking cessation programs and the design of an efficacy study that will be conducted to test the effects of the programs on smoking cessation. The computer-tailored e-health approaches are currently tested by Dutch smokers who are motivated to stop smoking. The tailored messages are the same in both conditions; the two conditions only differ in the mode of delivery. One experimental condition uses video-driven messages whereas the other condition uses text-driven messages. The two experimental conditions (video vs. text) will be compared with each other and with a control condition which receives general smoking cessation advice. Furthermore, the effects of the two different multiple tailored smoking cessation programs will be tested among smokers with a lower and higher socio-economic background.

The present study has several strengths. First, it addresses the efficacy of two computer-tailored programs by means of a strong experimental design. A randomized control trial was used to exclude possible biases with regard to assignment of respondents to the different conditions. Allocation of the different conditions was performed automatically with the Tailor Builder computer software.

Second, the intervention is one of the first programs to test video-based messages as a potential new communication strategy for LSES smokers. So far computer-tailored smoking cessation interventions have tended to rely on text-based messages. Several studies however suggest that videos may be more effective in attracting attention and stimulating comprehension, especially in LSES smokers [22].

Third, an important strength of our smoking cessation programs concerns the inclusion of multiple tailored feedback moments. Tailored information has been shown to be more likely to be read, remembered and considered personally relevant. Our study made use of a multiple computer-tailored smoking cessation intervention with different feedback moments. Although previous programs did focus on smoking cessation tailored messages, few of them provided smokers with multiple tailored feedback moments.

Finally, one strategy the study used to collect participants was via the GP setting. The GP is considered as an important access point to many smokers [46]. Advice from GPs is an effective way to help smokers to quit and is seen as valuable by the Dutch patient [47].

There may be limitations to the study. First, the multiple tailoring e-health programs could be too intensive and therefore lead to an increased drop-out rate. Furthermore, the program is only available on the internet, so smokers with no internet access were excluded from the study. Although 90% of Dutch households are equipped with internet access this could still result in selection bias [14].

Conclusion

A description was provided of the e-health smoking cessation intervention SteunbijStoppen.nl, which was developed especially to attract smokers with a lower socioeconomic status. The paper also explained the classification of the study into efficacy and cost-effectiveness of the two programs compared with a control condition and user evaluation. The study results reveal different working mechanisms of multiple computer-tailored smoking cessation interventions. The results will help us to gain more insight into effective strategies to target different subgroups and especially smokers with a lower social economic status. Finally, the results of the study will contribute to the development of future smoking cessation e-health programs. The different behavioural effects of the Steunbijstoppen.nl intervention will be published in other papers.

Acknowledgements

The study was funded by ZonMw, the Netherlands Organisation for Health Research and Development (grant number: 20011007).

Author details

[1]Department of Health Promotion, School for Public Health and Primary Care (CAPHRI), Maastricht University, P.O. Box, 6200 MD Maastricht, the Netherlands. [2]Department of Health Promotion and Health Education, Maastricht University, P.O. Box 616, 6200 MD Maastricht, the Netherlands. [3]Department of Psychology, Open University of the Netherlands, 6419 AT Heerlen, the Netherlands. [4]Department of General Practice, Maastricht University, P.O. Box 616 6200 MD Maastricht, the Netherlands.

Authors' contributions

HdV, CB and JM designed and wrote the original proposal. NES, HdV, CB and JM developed the smoking cessation intervention and execute the studies. NES significantly contributed to writing this paper, while HdV, CB and JM were involved in revising the manuscript critically. All authors read and approved the final version of the manuscript.

Competing interests

The authors declare that they have no competing interests.

Received: 11 October 2011 Accepted: 7 November 2011
Published: 7 November 2011

References

1. CDC: **The health consequences of smoking: a report of the Surgeon General.** Washington D.C.: Centers for Disease Control and Prevention; 2004.
2. Fiore MC, Jaen CR, Baker TB, Bailey WC, Benowitz N, Curry SJ, Dorfman SF, Froelicher ES, Goldstein MG, Healton CG, *et al*: **Clinical practice guideline: treating tobacco use and dependence.** *Public Health Service* 2008.
3. De Vries H, Brug H: **Computer-tailored interventions motivating people to adopt health promoting behaviours; introduction to a new approach.** *Patient Education and Counseling* 1999, **36**:99-105.
4. Lustria MLA, Cortese J, Noar SM, Glueckauf RL: **Computer-tailored health interventions delivered over the web:Review and analysis of key components.** *Patient Education and Counseling* 2009, **74(2)**:156-173.
5. Kreuter MW, Farwell D, Olevitch L, Brennan L, (eds.): **Tailored Health messages: Customizing Communication with Computer Technology.** Mahwah, New Jersey: Lewrence Erlbaum; 1999.
6. Smeets T, Brug J, De Vries H: **Effects of tailoring health messages on physical activity.** *Health Education Research* 2008, **23(3)**:402-413.
7. Oenema A, Brug J, Dijkstra A, Weerdt I, De Vries H: **Efficacy and use of an Internet-delivered Computer-tailored Lifestyle Intervention, Targeting saturated Fat Intake, Physical Activity and Smoking Cessation: A randomized Controlled Trial.** *Annual Behaviour Medicine* 2008, **35**:125-135.
8. Te Poel F, Bolman C, Reubsaet A, De Vries H: **Efficacy of a single computer-tailored e-mail for smoking cessation: results after 6 months.** *Health Education Research* 2009, **24(6)**:930-940.
9. Dijkstra A, De Vries H, Roijackers J, Van Breukelen G: **Tailored information to enhance quitting in smokers with low motivation to quit: three basic efficacy questions.** *Health Psychology* 1998, **17(6)**:513-519.
10. Dijkstra A, De Vries H, Roijackers MA: **Targeting smokers with low readiness to change with tailored and nontailored self-help materials.** *Preventive Medicine* 1999, **28**:203-211.
11. Dijkstra A, De Vries H: **Clusters of precontemplating smokers defined by the perception of the pros, cons, and self-efficacy.** *Addictive Behaviors* 2000, **25**:373-385.
12. Shahab L, Mc Ewen A: **Online support for smoking cessation: a systematic review of the literature.** *Addictive Behaviours* 2009, **104**:1792-1804.
13. Lancaster T, Stead L: **Self-help interventions for smoking cessation.** *Cochrane Database System Review* 2005, **3**.
14. CBS: **Centraal Bureau voor de Statistiek.**[http://www.cbs.nl].
15. Michie S, Jochelson K, Markham WA, Bridle C: **Low-income groups and behaviour change interventions: a review of intervention content, effectiveness and theoretical framework.** *Journal of Epidemiology Community Health* 2009, **63**:610-622.
16. Bell K, McCullough L, Greaves L, Mulryne R, Jategaonkar N, De Vries K: **The Effectiveness of National Health Service Intensive treatments for Smoking Cessation in England: a rapid review.** London, UK: National Institute for Clinical and Health Excellence; 2006.
17. Murphy PW, Andrew L, Chesson AL, Walker L, Arnold C: **Comparing the effectiveness of video and written material for improving knowledge among sleep disorders clinic patients with limited literacy skills.** *South Medical Journal* 2000, **93(3)**:297-304.
18. Brouwner C, Preloran M, Press N: **The effects of ethinicity, education and an informational video on pregnant women's knowledge and decisions about a prenatal diagnostic screening test.** *Patient Education and Counseling* 1996, **27**:135-146.
19. Haug S, Meyer C, Ulbricht S, Gross B, Rumpf HJ, John U: **Need for cognition as a predictor and a moderator of outcome in a tailored letters intervention for smoking cessation.** *Health Psychology* 2010, **29(4)**:367-373.
20. Cacioppo J, Petty R, Feinstein J: **Dispositional differences in cognitive motivation: The life and times of individuals varying in need for cognition.** *Psychol Bulletin* 1996, **199(2)**:197-253.
21. Mayer R, Massa L: **Three facets of visual and verbal learners: cognitive ability, cognitive style and learning preference.** *Journal of Education Psychology* 2003, **95(4)**:833-846.
22. Nielsen E, Sheppard MA: **Television as a patient education tool: A review of its effectiveness.** *Patient Education and Counseling* 1988, **11(1)**:3-16.
23. Salomon G: **Television Is Easy and Print is Tough: The differences investment of mental effort in learning as a function of perceptions and attributions.** *Journal of Education Psychology* 1984, **76(4)**:647-658.
24. Tversky B, Morrison J, Betrancourt M: **Animation: Can it facilitate?** *International journal of human computer studies* 2002, **57**:247-262.
25. De Vries H, Kremers SP, Smeets T, Brug J, Eijmael K: **The effectiveness of tailored feedback and action plans in an intervention addressing multiple health behaviours.** *American Journal of Health Promotion* 2008, **22(6)**:417-425.
26. Smit ES, Hoving C, De Vries H: **The PAS study: A randomized controlled trial evaluating the effectiveness of a web-based multiple tailored smoking cessation programme and tailored counselling by practice nurses.** *Contemporary Clinical Trials* 2010, **31**:251-258.
27. OSE: **OverNite Software Europe BV.**[http://http//:www.ose.nl].
28. Hughes JR, Keely J, Naud S: **Shape of the relapse curve and long-term abstinence among untreated smokers.** *Addiction* 2003, **99**:29-38.
29. Cole S, Williamson GM: **Development and validation of a Rasch-derived CES-D short form.** *Psychol Assessment* 2004, **61**:360-372.
30. Euro EG: **A new facility for the measurement of health related quality of life.** *Health Policy* 1990, **16(3)**:199-208.
31. Cole JC, Rabin AS, Smith TL, Kaufman AS: **Development and validation of a Rasch-Derived CES-D short form.** *Psychological Assessment* 2004, **16**:360-372.

32. Cacioppo JT, Petty RE: **The need for cognition.** *Journal of Personality and Social Psychology* 1982, **42(116-131)**.
33. Heatherton TF, Kozlowski LT, Frecker RC, Fagerstroem KO: **The Fagerstrom Test for Nicotine Dependence: a revision of the Fagerstrom Tolerance Questionnaire.** *British Journal of Addiction* 1991, **86**:1119-1127.
34. Verplanken B, Orbell S: **Reflections on past behavior. A self-report index of habit strength.** *Journal of Applied Social Psychology* 2003, **33**:1313-1330.
35. Dijkstra A, De Vries H, Bakker M: **Pros and cons of quitting, self-efficacy, and the stages related to smoking cessation.** *Journal of Consulting and Clinical Psychology* 1996, **1996(64)**:758-763.
36. Dijkstra A, De Vries H: **Self-efficacy with regard to different tasks in smoking cessation.** *Psychology and Health* 2000, **14(4)**:501-511.
37. Hoving C, Mudde AN, De Vries H: **Predictors of smoking relapse in a sample of Dutch adult smokers: the roles of gender and action plans.** *Addictive Behaviours* 2006, **31**:1177-1189.
38. Baylor AL: **Promoting motivation with virtual agents and avaters: role of visual presence and appearance.** *Philosophical transactions of the Royal Society* 2009, **364**:3559-3565.
39. Bailenson JN, Blascovich J, Guadagno RE: **Self-representations in immersive virtual environment.** *Journal of Applied Social Psychology* 2008, **38**:2673-2690.
40. Gwaltney CJ, Metrik J, Kahler CW, Shiffman S: **Self-efficacy and smoking cessation: A meta-analysis.** *Psychology of Addictive Behaviours* 2009, **23(1)**:56-66.
41. Gwaltney CJ, Metrik J, Kahler CW, Shiffman S: **Dynamic Self-Efficacy and Outcome Expectancies: Prediction of Smoking Lapse and Relapse.** *Journal Abnormal Social Psychology* 2005, **114(4)**:661-675.
42. Elfeddali I, Bolman C, De Vries H: **SQ4U- a computer tailored smoking relapse prevention program incorporating planning strategies and multiple feedback moments after the quit-attempt: development and design protocol.** *submitted* 2011.
43. Gollwitzer P: **Implementation intentions. Strong effects of simple plans.** *American Psychologist* 1999, **54(7)**:439-503.
44. Mudde AN, Willemsen MC, Kremers S, De Vries H: **Meetinstrumenten voor onderzoek naar roken en stoppen met roken. (Measurements from studies about smoking and smoking cessation).** *Stivoro* 2006.
45. Velicer WF, Prochaska JO: **A comparison of four self-report smoking cessation outcome measures.** *Addictive Behaviours* 2004, **29(1)**:51-60.
46. Van den Berg M: **De werkdruk van de huisarts (The GP's work-related pressure).** *Medisch Contact* 2003, **58**:1054-1056.
47. Wensing M, Vedsted P, Kersnik J, Peersman W, Klingenberg A, Hearnshaw H, Hjortdahl P, Paulus D, Kuenzi B, Mendive J, *et al*: **Patient satisfaction with availability of general practice: an international comparison.** *International Journal for Quality in Health Care* 2002, **14**:111-118.

Pre-publication history

The pre-publication history for this paper can be accessed here:
http://www.biomedcentral.com/1471-2458/11/847/prepub

doi:10.1186/1471-2458-11-847
Cite this article as: Stanczyk *et al.*: Study protocol of a Dutch smoking cessation e-health program. *BMC Public Health* 2011 **11**:847.

Submit your next manuscript to BioMed Central and take full advantage of:

- **Convenient online submission**
- **Thorough peer review**
- **No space constraints or color figure charges**
- **Immediate publication on acceptance**
- **Inclusion in PubMed, CAS, Scopus and Google Scholar**
- **Research which is freely available for redistribution**

Submit your manuscript at
www.biomedcentral.com/submit

BioMed Central
